# Frequency and Molecular Characterization of *Staphylococcus aureus* from Placenta of Mothers with Term and Preterm Deliveries

**DOI:** 10.3390/life12020257

**Published:** 2022-02-09

**Authors:** Hafiz Muhammad Umer Farooqi, Kyung-Hwan Kim, Farzana Kausar, Javed Muhammad, Habib Bukhari, Kyung-Hyun Choi

**Affiliations:** 1 Department of Mechatronics Engineering, Jeju National University, Jeju-si 63243, Korea; umerfarooqi@jejunu.ac.kr (H.M.U.F.); kyunghwankim@jejunu.ac.kr (K.-H.K.); 2 Department of Biosciences, COMSATS University, Islamabad 45550, Pakistan; 3 National Control Laboratory for Biologicals, Drug Regulatory Authority of Pakistan, Islamabad 44090, Pakistan; 4 Department of Plant Sciences, Quaid-i-Azam University, Islamabad 45330, Pakistan; kausarfarzana4915@gmail.com; 5 Department of Microbiology, University of Haripur, Haripur 22621, Pakistan; javed.muhammad@uoh.edu.pk

**Keywords:** placenta, neonates, term, preterm, methicillin-resistant *Staphylococcus aureus* (MRSA), *Staphylococcus aureus *(*S. aureus*)**, antimicrobial activity

## Abstract

Globally, prematurity is the leading cause of neonatal mortality (babies in the first four weeks of life) and now the second leading cause of mortality after pneumonia in children under age five. The neonatal gut microbial colonization is crucial in the human life cycle. Placental microbiota transmits from the gut microbiota plays a significant role in association with kinship. Simultaneously, this transition is being made from mother to infant. This comparative study explored the diversity of microbiota associated with term and preterm neonates by evaluating the placental samples. The study found that 16/68 (23.5%) full-term placental samples were positive for *S. aureus;* on the other hand, 4/16 (25%) preterm placental samples confirmed culture growth for *S. aureus.* Antimicrobial susceptibility patterns showed that *Staphylococcus* *aureus* (*S. aureus*) isolates from both types of samples were resistant to Ofloxacin, Trimethoprim-sulfamethoxazole, Oxacillin, and Cefoxitin. However, Methicillin-Resistant *Staphylococcus aureus* (MRSA) detection was 43.75% in full-term and 75% in preterm placental samples. Moreover, two isolates were positive for both *mecA* and *PVL* virulent genes, and the rest were positive only for the *mecA* gene. Interestingly few isolates lacked both characteristic MRSA genes, *mecA* and *PVL*. Notably, resistances were more inclined towards preterm samples for antimicrobial susceptibility and MRSA screening. It may be concluded that there is a significant presence of *S. aureus* in the placenta of mothers with term and preterm deliveries which might be responsible for preterm deliveries. Therefore, judicious use of antibiotics during pregnancies may help prevent preterm births.

## 1. Introduction

A healthy pregnancy is associated with a unique host environment, dynamic state of altered anatomy, physiology, and immune function modeling. Limited data suggest how the microbiota is being shaped over time with the crucial outcome of term and preterm deliveries. The microbiota has also been a vital player in health and disease states, including nutrient acquisition, immune modeling, and subsequent protection from microbial colonization [1,2]. However, preterm birth, i.e., before 37 weeks of gestation, is one of the leading causes of neonatal death [3]. The role of microbiota in term and preterm birth is poorly understood. Based on the previous literature, infections and associated inflammation are the vital cause of spontaneous preterm deliveries [4]. These are the most common bacterial infections rising to the amniotic cavity from the vagina and cervix [5]. Therefore, acquisition and diversity of the gut microbiota in neonates have been the subject of several studies, but their association with preterm birth is less studied. It is reported that abnormal intestinal colonization during the first weeks of life may alter functions such as barrier integrity and the nutritional and immunological behavior of the host and subsequently increase susceptibility to infectious disease. The placenta plays a pivotal role in nourishing the fetus and provides humoral immunity to the newborn [6,7]. The placenta harbors the intracellular bacteria that synthesize a wide variety of cytokines, and it may also house pathogenic bacteria and eventually preterm birth (PTB) [8,9].

Microorganisms recovered from the uterine cavity (including the amniotic fluid) following PTB include *Chlamydia trachomatis, Trichomonas vaginalis, Ureaplasma urealyticum, Streptococcus agalactiae*, *Escherichia coli*, as well as various anaerobes [10]. Some of these, including species of *Fusobacterium* and *Streptococcus*, are also found in the oral cavity [11]. Detection of bacteria in amniotic fluid correlates to histological inflammation; higher grades of histological lesions are associated with increasing colony counts of bacteria in amniotic fluid and with “high-virulence” bacteria in amniotic fluid. However, in approximately 30% of preterm premature rupture of membranes (PPROM) cases, the recovery of bacterial organisms does not correlate with inflammatory changes found during histological chorioamnionitis [12].

The incidence of *Staphylococcus aureus (S. aureus)* infections is increasing in postpartum-pregnant and pregnant women and neonatal intensive care units (NICUs) and healthy neonates [13]. Most of the cases are due to rising rates of methicillin-resistant *Staphylococcus aureus* (MRSA), specifically community-associated (CA)–MRSA, which are involved in causing infections in patients where traditional risk factors are not present [14,15]. *S. aureus* infections are more common among individuals who harbor *S. aureus* in the anterior nares [16,17]. Reportedly, the vagina of 4–22% of pregnant women is colonized by *S. aureus*, out of which MRSA colonizes 0.5–10% of the pregnant women’s vagina [18].

The development of antibiotic resistance in *S. aureus* has been long known since mid-1940. The mechanism of penicillin resistance in this bacterium was determined due to the presence of Penicillin hydrolyzing enzymes, named penicillinase [19]. Resistance to Penicillin increased until 1960 when semi-synthetic analogs of Penicillin were introduced [20]. However, even the development of these analogs, such as methicillin, could not stop *S. aureus* from developing resistance. Infection and sooner Methicillin-resistant *S. aureus* (MRSA) were reported in many cases. The methicillin resistance was due to acquiring a genomic island carrying methicillin resistance determinant, *mecA*. Since then, MRSA has been the most common cause of human, community, and livestock-associated infections worldwide. The rapid development of resistance and continuous evolution of this pathogen has acquired unique resistance mechanisms, such as developing methicillin resistance determinants coding for an alternative penicillin-binding protein with much-reduced susceptibility to most beta-lactams antibiotics class [21]. Coagulase-negative *S. aureus* and Coagulase-positive *S. aureus*, including community-associated MRSA, are involved in late-onset nosocomial sepsis in the neonatal period. In addition, reports of early-onset maternal-fetal infections with *S. aureus* are found. A single-center study described seven premature infants with congenital *S. aureus* infection, with both blood and amniotic fluid cultures being positive. In most cases, risk factors included antenatally invasive procedures (amniocentesis or amnioinfusion) performed within a day of delivery [22].

In this study, considering the importance of placental microbiota, placental specimens were collected from the women with full-term and preterm deliveries. The microbiological cultures were performed for the isolation of *S. aureus*. Biochemical analysis of the colonies was performed for the identification of *S. aureus*. Antimicrobial susceptibility testing was carried out to exclude the resistant species of *S. aureus* from antimicrobial susceptible isolates. The isolates’ molecular analysis and plasmid profiling were also performed to charac-terize the *S. aureus* isolated from the placental specimens collected from the women with full-term and preterm deliveries. We have analyzed the prevalence of *S. aureus* in the placenta of mothers with term and preterm deliveries. Moreover, the resistance pattern of the isolated *S. aureus* was determined as well. The study was intended to reveal the possible correlation between the presence of *S. aureus* in the placenta and the nature of delivery.

## 2. Materials and Methods

### 2.1. Sample Collection

A cross-sectional study was conducted where 84 placenta were collected from patients attending the Pakistan Institute of Medical Sciences (PIMS), Islamabad, Pakistan, from January to October. Trained obstetricians performed the placental sample collection by following the institutional biosafety and biosecurity committee guidelines, and placentas were collected in autoclaved containers using powder-free sterile gloves and dissection instruments, which took place in a special cabin in operation theater equipped with a UV light source for surface decontamination. Four 0.5 cm × 0.5 cm cross-sectional placental tissue samples were excised, each at 3.5 cm from the cord insertion site [23]. One sample was immediately transferred into a sterile tube containing RNA later, while another biopsy was used for heavy metal analysis. One biopsy was homogenized and used for direct bacterial culture, and another sample was stored at −20C to extract the DNA. The samples collected in sterile falcon tubes were immediately brought to Microbiology and Immunology laboratory, COMSATS University Islamabad, for microbiological and molecular biological tests and processed within 1 h of collection. Considering the infectious property of *S. aureus*, all the samples were processed in biosafety level 2 (BSL-2) cabinets.

### 2.2. Culturing on MSA Media

For the selective growth of *S.* *aureus*, all the samples were directly cultured on Mannitol Salt agar (MSA) (Oxoid). Following the overnight incubation at 37 °C, the isolates were subjected for microscopic and biochemical characterization using catalase, oxidase, mannitol fermentation, and coagulase tests using the principles of Karmakar et al.

### 2.3. Antimicrobial Activity by Disc Diffusion Method

The antimicrobial susceptibility testing of the isolates was carried out using modified Kirby-Bauer disk diffusion method on Mueller-Hinton agar as recommended by Clinical Laboratory Standards Institute’s (CLSI) guidelines, 2020 using M2-A9 M7-A7 standards. The tested antibiotic disks were selected per CLSI 2020 and obtained from Oxoid, England, including Ofloxacin, Doxycycline, Oxacillin, Cefoxitin, Clindamycin Vancomycin, Trimethoprim/Sulfamethoxazole, and Linezolid. All the *S. aureus* isolates were screened for methicillin resistance by disc diffusion (6 µg/mL oxacillin) on Mueller-Hinton agar (CLSI, 2020).

### 2.4. 16S rRNA Analysis of Isolates

DNA was extracted by boiling method given by Miller protocol [24]. Isolated DNA was used as a template to amplify DNA using universal primers 27f having sequence 5’-AGAGTTTGATCCTGGCTCAG-3’ and reverse primer 1522r having sequence 5’AAGGAGGTGATCCA(AG)CCGCA-3’. The 20 µL reaction mixture was prepared using 2 µL DNA, 2.5 µL Taq buffer, 2 µL MgCl_2_, 0.4 µL dNTPs, 1µL each forward and reverse primers, 10.6 µL H_2_O, and 0.5 µL Taq polymerase. The amplification was performed in a T-Personal Thermal Cycler (Biometra, MD, USA) with an initial denaturation step of 98 °C for 1 min followed by 30 cycles of 98 °C for 10 s, 52 °C for 30 s, and 72 °C for 30 s and a final extension at 72 °C for 10 min. PCR products were purified by using a purification kit and sequenced.

### 2.5. Molecular Identification of Virulent Genes

The optimized multiplex PCR components were used as follow: 2 µL of template DNA which was prepared by the boiling method in a 25 µL final reaction volume containing 1 µL of each forward and reverse primers for *PVL* and *mecA* genes, 2.5 µL MgCl_2_, 2.5 µL PCR buffer, 0.5 µL dNTPs, 0.5 µL Taq polymerase enzyme and 13 µL deionized water. The PCR conditions set for the first-round process was 94 °C for 10 min, and then second round was set as ten cycles of 94 °C for 45 s, 55 °C for 45 s, and 72 °C for 75 s and 25 cycles of 94 °C for 45 s, 50 °C for 45 s, and 72 °C for 75 s. The PCR products were analyzed on 2% agarose gel. Images of the PCR product were analyzed through a UV illuminator and gel doc machine.

### 2.6. Plasmid Profiling

The pure culture of *S. aureus* was inoculated into Luria–Bertani (LB) broth with Ampicillin and incubated overnight at 37 °C. The plasmid was extracted using the Five-Minute Plasmid Miniprep Kit GenElute™ (PLN70-1KT) [25]. Plasmid DNA was separated by horizontal electrophoresis in 1.5% agarose gel and visualized under the UV transilluminator. The molecular weight of the unknown plasmid DNA was determined based on its mobility through agarose gel compared to the molecular weight marker. Ethical approval was taken from the Department of Microbiology & Immunology, Comsats University Islamabad, Pakistan.

## 3. Results

### 3.1. Distribution of Samples

**Figure 1:**
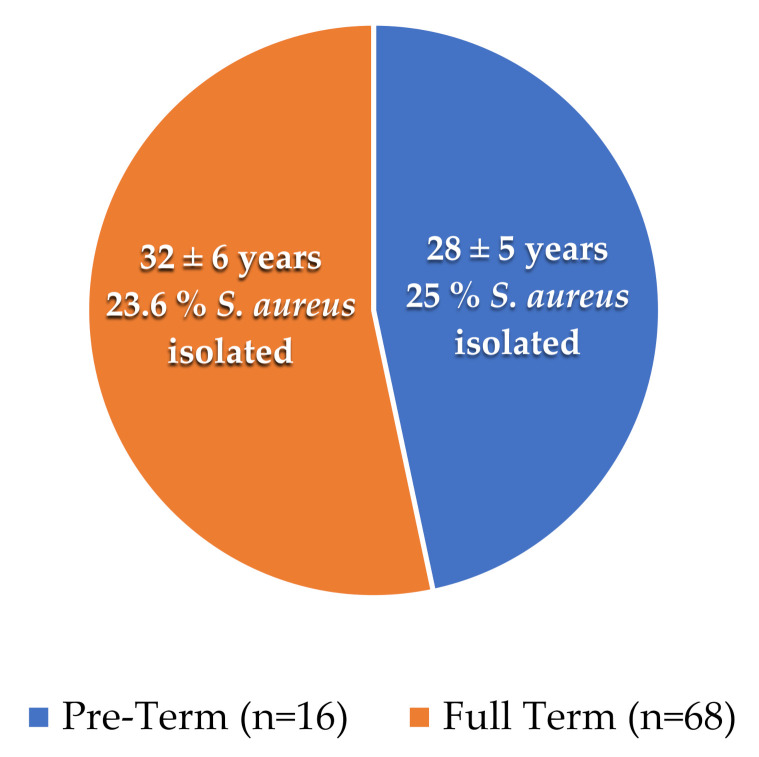
Mean age and number of isolates from full-term and preterm samples: The pie chart shows the presence of S. aureus percentages in tested samples. The orange color represents the full-term cases, while the blue represents the preterm cases.

A total of 68 full-terms and 16 preterm placental samples were collected with the mean age of (32 ± 6 years) and (28 ± 5 years), respectively. We observed that 04 and 16 samples were positive for *S. aureus* from the placenta of preterm and full-term patients, respectively, as shown in Table 1 and Figure 1.

### 3.2. S. aureus Culturing

*S. aureus* exhibited yellow-colored colonies on Mannitol Salt Agar (MSA). In addition, gram-positive round chained/grape-like structured colonies were observed under a compound light microscope. All the isolates were subjected to a catalase test showing effervescence upon contact with the catalase reagent. For the coagulase test confirmation, the coagulum production indicated the ability of the organism to coagulate the plasma, terminally confirming the isolates as *S. aureus*.

### 3.3. Antimicrobial Activity by Disc Diffusion Method

**Figure 2:**
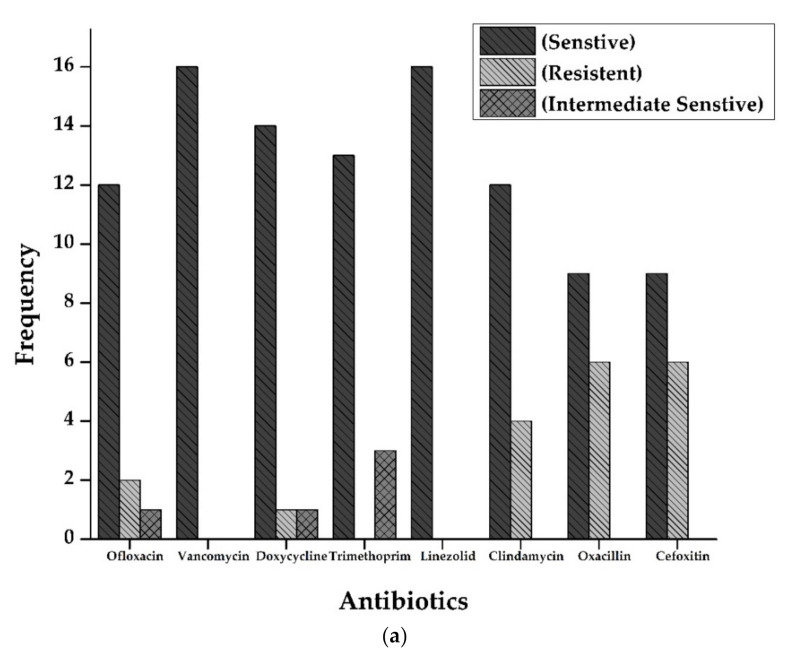

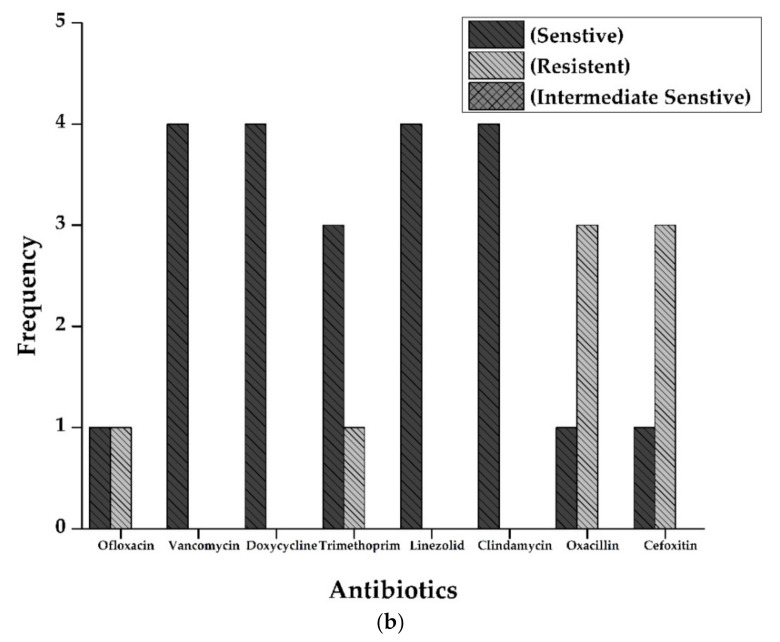
(**a**): Antibiogram of *Staphylococcus aureus* for Full-Term births. The resistance showed by *S. aureus* isolates to various antibiotics tested. *Y*-axis represents the number of strains showing resistance to various antibiotics tested. Vancomycin and Linezolid showed maximum sensitivity (16 out of 16 isolates), followed by Doxycycline (14), Trimethoprim (13), Ofloxacin, and Clindamycin (12 each of 16 samples). Resistance pattern shows that Oxacillin and Cefoxitin were the most resistant (6 out of 16 samples), followed by Clindamycin (4 out of 16). (**b**): Antibiogram of *Staphylococcus aureus* for preterm births. The resistance showed by *S. aureus* isolates to various antibiotics tested. *Y*-axis represents the number of strains showing resistance to multiple antibiotics tested. All the isolates were sensitive against Vancomycin, Doxycycline, Linezolid, and Clindamycin. At the same time, three isolates were resistant against Oxacillin and cefoxitin (3 out of 4 isolates).

**Figure 3:**
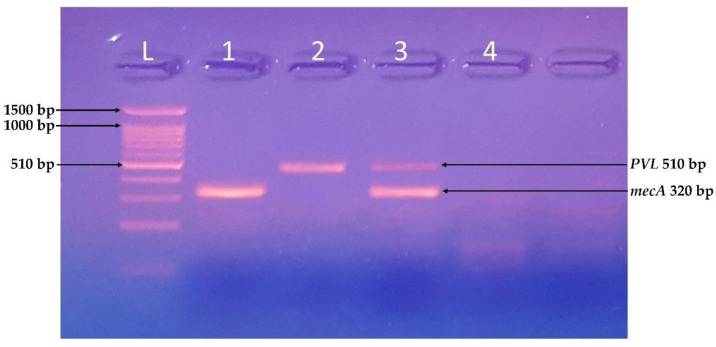
Identification of *mecA* and *PVL* genes. PCR-based identification of virulent genes of S. aureus using a ladder of 100 bp, Samples 1 and 3 were positive for *mecA* gene (320 bp), and samples 2 and 4 were positive for *PVL* gene (510 bp).

Antibiotic susceptibility patterns of all isolates from full-term and preterm *S.* *aureus* exhibited resistance against Ofloxacin (15%), Doxycycline (5%), Oxacillin, and Cefoxitin (50%), and Clindamycin (20%). However, no resistance was observed in the case of Vancomycin, Trimethoprim/sulfamethoxazole, and Linezolid. The results are displayed in Figure 2 and Figure 3 and Table 2.

### 3.4. Molecular Identification of Virulent Genes

All the isolates were tested for the presence of virulence-related genes. The isolates A, B, E, G, I, and K were positive for the PCR based identification of virulent genes, i.e., *mecA* gene (320 bp), C and D were both *mecA* and *PVL* (510 bp) positive, whereas F, H, and J were negative for both genes.

## 4. Discussion

Antimicrobial resistance has become a global public health concern, particularly considering the development and spread of multidrug-resistant (MDR) pathogens and MDR genes [26]. Therefore, it becomes critical to analyze the prevalence of MDR pathogens in the environment, in animals, and various human tissues. In this study, we have determined the presence of *S. aureus* in the placenta of mothers with term and preterm deliveries. Moreover, most isolated strains showed significant resistance to various clinically vital antibiotics. Such pathogenic strains in the placenta with multiple resistance mechanisms against clinically necessary antibiotics might be associated with preterm deliveries. Recent studies showed that *S. aureus* induced pro-inflammatory cytokines, the main contributing factor of preterm deliveries [26]. The MRSA load can be a leading cause of preterm births with fewer than 32 weeks gestation [27]. Furthermore, *S. aureus* is resistant to all the β-lactam drugs available now. Thus, the presence of drug-resistant *S. aureus* corresponds to a paradigmatic pathogen.

The present study comprises a 4/5th proportion of full-term births and 1/5th of preterm births. However, an almost equal proportion of the *S. aureus* isolates were obtained from both categories of participants, which is consistent with a study reported by Gitman et al., where 1/4th proportion of the bacterial population was isolated from the placenta delivered by cesarean section. The study also revealed that preterm labor had higher rates, decreasing with increasing gestational age. Thus, the study’s findings are comparable with the present study [28]. In the present study, 7/18; (39%) term positive and 3/4; (75%) of preterm isolates were tested for MRSA. The results follow the case study by Khan et al., 2020 where MRSA has also been reported in the placenta of preterm babies [9]. Hence, placental isolation of MRSA from preterm patients is highly significant.

Antimicrobial resistance patterns of all isolates from term and preterm *S. aureus* were OFX (15%), DOX (5%), OX and CEF (50%), Clindamycin (20%). However, low to zero percent resistance was observed in the case of VAN, SXT, and LZD. A study by Bauters et al., in 2021 also reported similar results in comparison to the present study, showing that out of a total of 906 *S. aureus* isolates, 250; (31.1%) and 39; (37.9%) were found to be methicillin-resistant [29]. Atolagbe et al., 2021 reported (99.6%) resistance against Penicillin, (93.6%) against Ampicillin, and (63.2%) against Gentamicin. However, 100% sensitivity was observed in the case of Vancomycin [30].

In the present study, out of total isolated bacterial strains, the *mecA* gene was detected only in two isolates, and no *PVL* gene belonged to full-term samples. However, in the case of preterm isolates, four *mecA* and two *PVL* genes were isolated. Furthermore, the study of Skiba et al., 2021, reported a relatively higher percentage (89%) of the *mecA* gene prevalence in MRSA isolates in preterm patients [31]. Interestingly two isolates were negative for both *PVL* and *mecA* gene, suggesting the poor association of isolated MRSA with hospital-acquired MRSA.

This study demonstrated the prevalence of potentially pathogenic *S. aureus* isolates from the placenta of mothers with term and preterm deliveries. The presence of pathogenic organisms in the placenta and their resistance to various clinically significant antibiotics raise questions on the possible association of the mentioned pathogen with preterm deliveries and the prophylaxis process. Multiple resistance mechanisms render a pathogen hard to tackle and limit the choice of already available antibiotics. Our study further elaborates that the mother gut microbiome originated microbiota colonized on the placenta might have consequences on fetal development and the nature of the delivery.

## 5. Conclusions

The significant presence of *S. aureus* in the placenta from women with term and preterm births indicates a considerable correlation between placenta harboring *S. aureus* species and maybe a clue to the onset of preterm delivery. Furthermore, the drug-resistance pattern suggests that antibiotic resistance is found in bacteria isolated from the placenta of women with preterm births, making them more prone to secondary infections and preterm labor. The lack of MRSA characteristic genes, i.e., *mecA* and *PVL*, in the isolates from the placenta of the women with preterm deliveries forecasted the novelty of the isolates and excluded them from the environmental contaminants. Further, this necessitates better clinical management of infections for pregnant women to reduce the rate of preterm deliveries in low-income countries.

## Figures and Tables

**Table 1:** Distribution of Full-Term and Preterm isolates.

Source	Total Samples	Positive Samples
Full-Term Babies	68	16 (23.5%)
Preterm Babies	12	4 (33.3%)

**Table 2:** Frequency of MRSA among full-term and preterm samples.

Source	Total *S. aureus*	MRSA Positive
Full-Term	16	7 (43.7%)
Preterm	4	3 (75.0%)

## Data Availability

The data supporting this study are available from the corresponding author upon reasonable request.
